# Eosinophilic lingual tonsillitis induced by sublingual immunotherapy: A case series

**DOI:** 10.1002/ccr3.3123

**Published:** 2020-08-06

**Authors:** Carlo Cavaliere, Paolo Luperto, Michele Gnesutta, Cristoforo Incorvaia, Davide Rosati, Simonetta Masieri

**Affiliations:** ^1^ Department of Oral and Maxillofacial Sciences Sapienza University Rome Italy; ^2^ Section of Otolaryngology ASL BR1 Brindisi Italy; ^3^ Department of Sense Organs Sapienza University Rome Italy; ^4^ Cardiac/Pulmonary Rehabilitation ASST Pini/CTO Milan Italy; ^5^ Section of Otolaryngology and Cervico‐Facial Surgery San Camillo de Lellis Hospital Rieti Italy

**Keywords:** adverse reactions, allergic rhinitis, eosinophils, lingual tonsil, sublingual immunotherapy

## Abstract

The new observation of eosinophilic lingual tonsillitis as a possible adverse reaction to sublingual immunotherapy is likely to stimulate new investigations aimed at improving the understanding of the mechanisms of biodistribution of extracts placed at the sublingual level.

## Graphical abstract

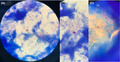

## 1 INTRODUCTION

Allergic rhinitis (AR) is a widespread disease, with a prevalence estimated from 20% to 40%.^1^ According to the Allergic Rhinitis and its Impact on Asthma (ARIA) document, allergen immunotherapy should play an essential role in the treatment of AR.^2^ In particular, sublingual immunotherapy (SLIT) is progressively more used worldwide and new high‐quality products have been registered as drugs for respiratory allergy by the European Medicine Agency (EMA) and the Food and Drug Administration (FDA).^3^ The overall safety of SLIT is superior to that of subcutaneous immunotherapy in terms of systemic adverse events; no fatality has been reported, and most adverse events are confined to the site of administration.

Recently, it has been reported a possible role for the lingual tonsil in the biodistribution of the allergen extracts administered by the sublingual route.^4^ In this case series, we describe three clinical scenarios that, for the first time, suggest the onset of lingual tonsillitis during SLIT; this could explain common local side effects like throat irritation.

## 2 CASE REPORTS

### 2.1 Case 1

**Figure 1:**
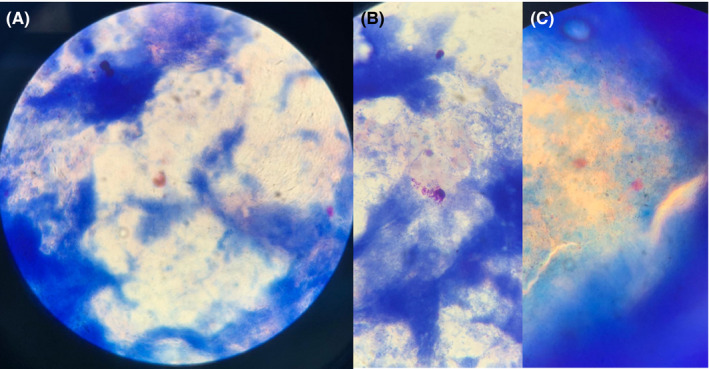
A, A degraded eosinophilic cell, with the characteristic presence of mixed bacterial flora and spores of the base tongue. B, Two degranulated mast cells. C, Abundant eosinophilic granules resulting from cell degeneration

17‐year‐old female. Affected by seasonal rhinitis, the patient had prick tests and in vitro IgE tests positive for grass pollen. She started immunotherapy with Phleum pratense pollen SLIT tablets (Grazax) in 2017, but interrupted after two years because of product shortage and restarted in April 2019. However, she developed dyspnea and dysphonia, successfully treated with ebastine 20 mg, after the 5th administration. Nasal endoscopy showed a hypertrophic and cyanotic mucosa, and nasal and lingual cytology were positive for numerous degranulated eosinophils. SLIT was withdrawn with a rapid reversal of symptoms and cytological abnormalities, which maintained at controls after three months and one year. Figure 1 shows the pathological pictures concerning eosinophils and mast cells.

### 2.2 Case 2

30‐year‐old female. The patient was affected by seasonal rhinitis with positivity of prick tests and in vitro IgE for grass pollen. Immunotherapy with 5‐grass pollen SLIT tablets (Oralair, Stallergenes Greer) started in 2017. After the 7th administration, the subject manifested dyspnea, nausea, vomiting, and dizziness, successfully treated with betamethasone 3 mg and ebastine 20 mg. Physical examination revealed edema of the lingual tonsil and the pharyngolaryngeal mucous membranes. Lingual cytology was positive for abundant neutrophils and eosinophils. After treatment withdrawal, the clinical and cytological picture normalized and the evidence of normality of lingual cytology maintained at controls after 3 months and one year.

### 2.3 Case 3

26‐year‐old female. Prick test positive for dust mites. The patient started the immunotherapy with Staloral BM (Stallergenes Greer) mites tablet in 2017. After the 5th administration, she presented dyspnea, successfully treated with betamethasone 3 mg and ebastine 20 mg. Physical examination evidenced mild lingual edema (including lingual tonsil) and hypertrophy of the nasal mucosa. At lingual cytology, the presence of considerable neutrophilia and eosinophilia was seen. After stopping the treatment, there was the disappearance of eosinophils and a progressive decrease in the neutrophilic infiltrate. Lingual cytology showed no evidence of eosinophils after three months and one year.

## 3 METHODS

Lingual cytology was performed using a sterile disposable curette (nasal scraping—EP Medica, Ravenna, IT) commonly used for nasal cytology,^5^ but characterized by a slightly longer handle. The device was placed near the lingual tonsil through the mouth. The cytological sampling was carried out by lightly pressing the instruments on the mucosa of the lingual tonsil. The collected material was first transferred to a microscope slide and dried, then colored by May‐Grünwald‐Giemsa (MGG) staining to identify inflammatory cells better. The stained slide was read at optical microscopy, with a 1000x objective with oil immersion. Written informed consent was obtained from all patients.

## 4 DISCUSSION

Sublingual immunotherapy has an excellent safety profile, as shown initially in 2005,^6^ and recently confirmed.^7^ Local reactions in the site of contact with the administered allergen (including oropharyngeal and gastrointestinal mucosa) are the most frequent side effect. Our report is consistent with the literature since the reactions we observed in our three patients were local and did not represent a safety issue. Eosinophilic inflammation of lymphatic stations of the Waldeyer ring has been previously reported in subjects with allergic airways disease.^8^ However, this event has never been described at the lingual level in concomitance with the oromucosal side effects of a SLIT course, but our repeated observations hint that a systematic finding could be likely in subjects with local reactions. Moreover, in our opinion, this kind of reaction may help improve the knowledge in SLIT's mechanism of action, which has always been a source of debate, particularly to achieve a deeper understanding of the factors regulating biodistribution of the allergen extracts placed at a sublingual level.

The limit of our study is to have not performed a lingual tonsil biopsy to confirm the cytological findings. Unfortunately, a tonsil biopsy implies a high risk of bleeding and would have been difficult to justify in clinical practice. Future studies might consider the opportunity to perform a biopsy, but only after the approval by the ethics committee. Another limit of our observation consists in the lack of data on the presence of an eosinophilic inflammation before the SLIT and on the reoccurrence of inflammation at its reintroduction. On the other hand, the total disappearance following SLIT discontinuation in all three cases sounds quite unequivocal. It is known that eosinophils are part of the chronic inflammatory response to gastroesophageal acid reflux (GERD),^9^ but they are mainly expressed in the esophageal mucosa, and none of our patients reported a diagnosis of GERD.

The eosinophilic inflammation caused by an allergen during its transit to the gastrointestinal tract strongly suggests its contact with an essential component of the Waldeyer ring. The discovery of a new entity, that is, the eosinophilic lingual tonsillitis during SLIT, and the involvement of the lingual tonsil in the allergen's crossing to the immune system may open the door to a further scientific advance in understanding this mechanism, which is essential to recognize the biodistribution of extracts placed at a sublingual level.

## CONFLICT OF INTEREST

CC, LP, GM, RD, MS declare that they have no conflicts of interest. IC has been a scientific consultant for Bayer S.p.A. and Stallergens Srl.

## AUTHOR CONTRIBUTIONS

CC, GM, MS: contributed to the design and implementation of the research. CC, LP, GM, RD, MS: developed the performed the measurements. CC, IC, MS: contributed to the analysis of the results and to the writing of the manuscript.
